# Supplementary material for: Ribosomal DNA copy number is associated with body mass in humans and other mammals
Source: Nat Commun. Author manuscript; Available in PMC 2024 Jun 13. (PMC11169392; doi:10.1038/s41467-024-49397-5)
Supplement: Supplementary Information [file EMS196930-supplement-Supplementary_Information.pdf]

Supplementary Material

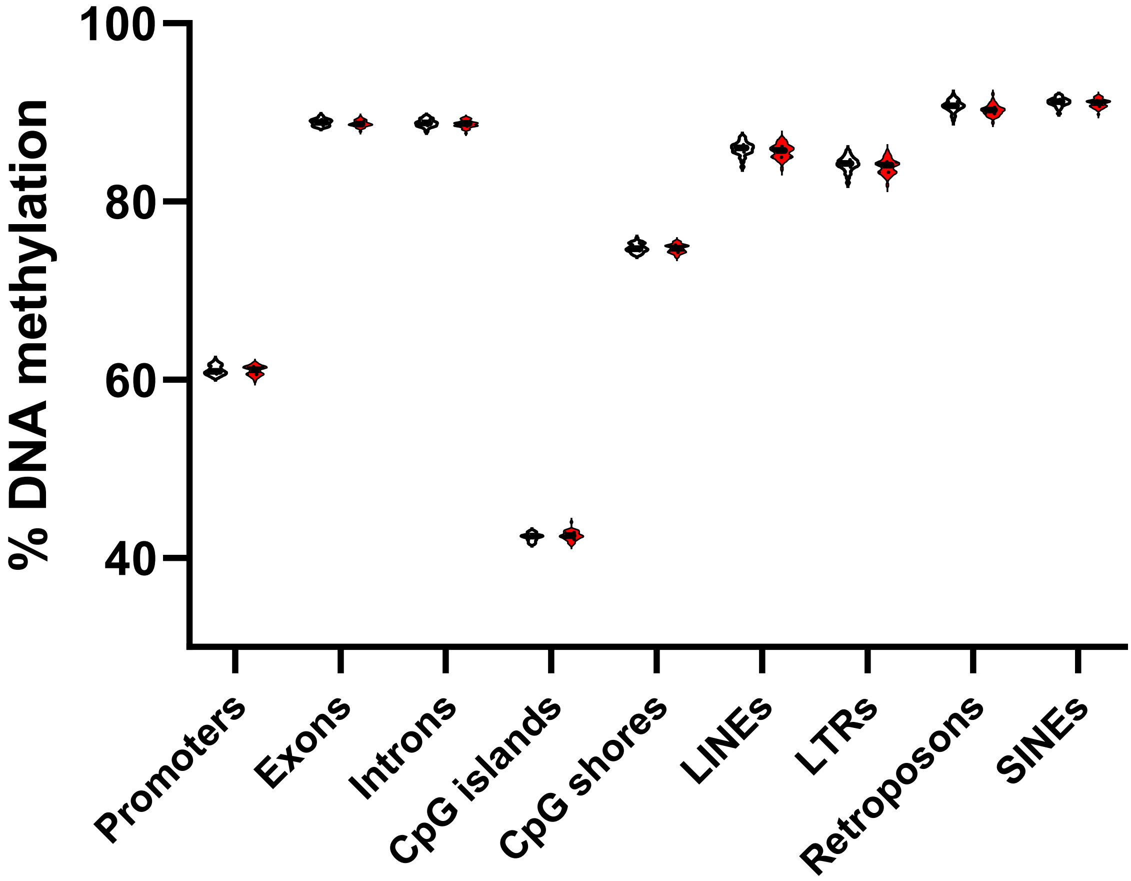

Figure S1. Methylation of classes of genomic features in lean and obese groups of the discovery cohort. Mean methylation was calculated for all CpG sites captured within reads mapping to the above specific annotations. No features were considered to be differentially methylated between lean and obese groups with Multiple Mann-Whitney tests (P_{adj} <0.01). The lean group (from n=31 individual males) is represented by clear and obese by red violin (from n=32 individual males) plots.

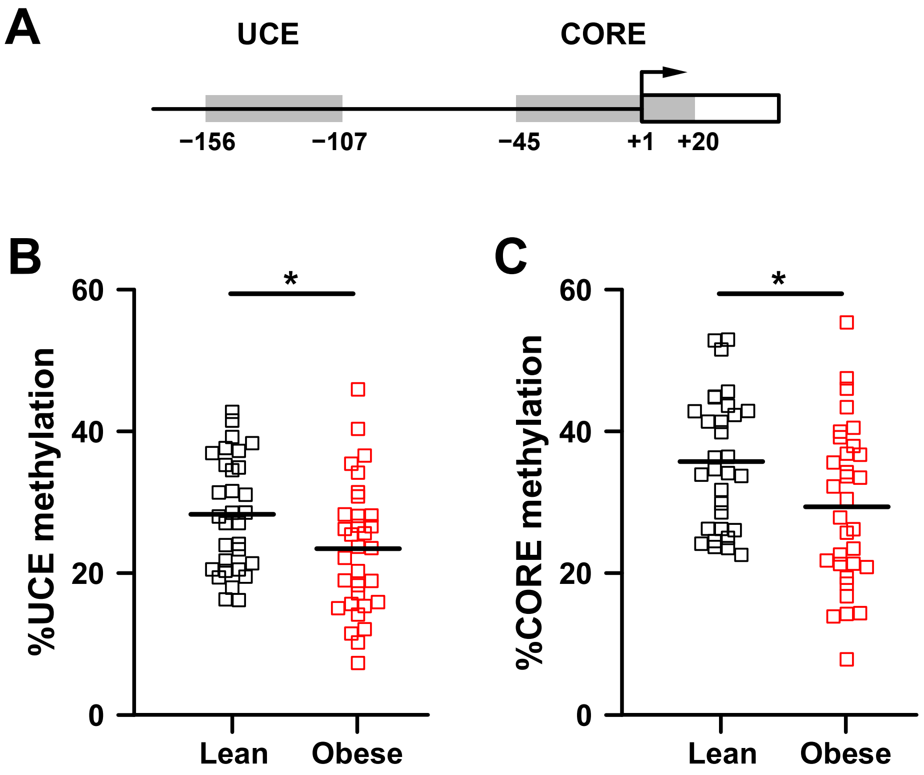

Figure S2. Methylation of core regulatory units within the rDNA promoter. (A) Positions of the upstream control element (UCE) and core promoter (CORE) relative to the transcriptional start site (+1) are indicated. **(B)** Methylation of the UCE is significantly lower in the obese (n=32 individual males) compared to the lean (n=31 individual males) group (two sided Mann-Whitney test, p=0.0213). **(B)** Methylation of the CORE is significantly lower in the obese (n=32 individual males) compared to the lean (n=31 individual males) group (two sided Mann-Whitney test, p=0.0177).

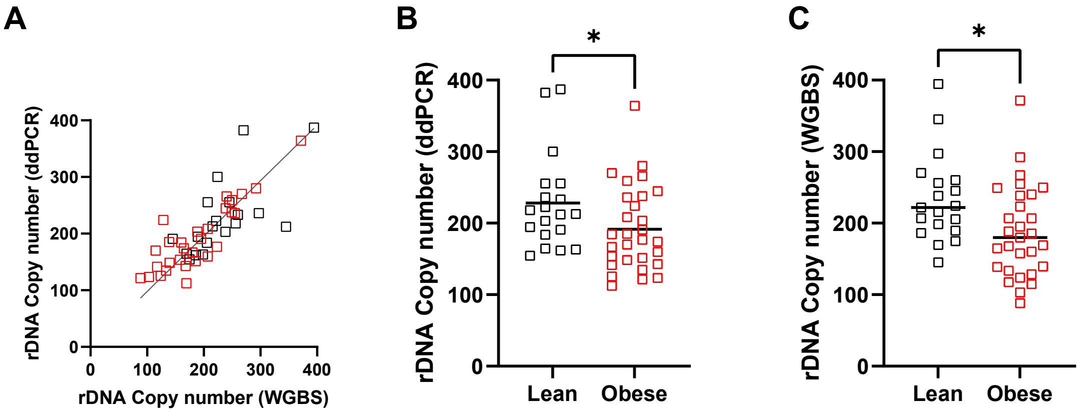

Figure S3. Validation of WGBS rDNA CN assessment by a sequencing independent method- digital droplet PCR (ddPCR). A) rDNA CN determined by WGBS is correlated with rDNA CN assessed by ddPCR (Spearman r = 0.8135, p<0.0001, n = 48 individual males). **B)** rDNA CN in the obese group is lower when assessed using ddPCR (p = 0.0492, two-sided Mann-Whitney test). **C)** rDNA CN in the obese group is lower when assessed using WGBS (p = 0.0119, Mann-Whitney test). Throughout lean (BMI<25 kg/m^2, n=19 individual males, black) or obese (BMI>30 kg/m^2, n=29 individual males, red). Sample number is restricted due to not having sufficient amount of sample remaining to perform ddPCR validation for all individuals that passed the WGBS QC.

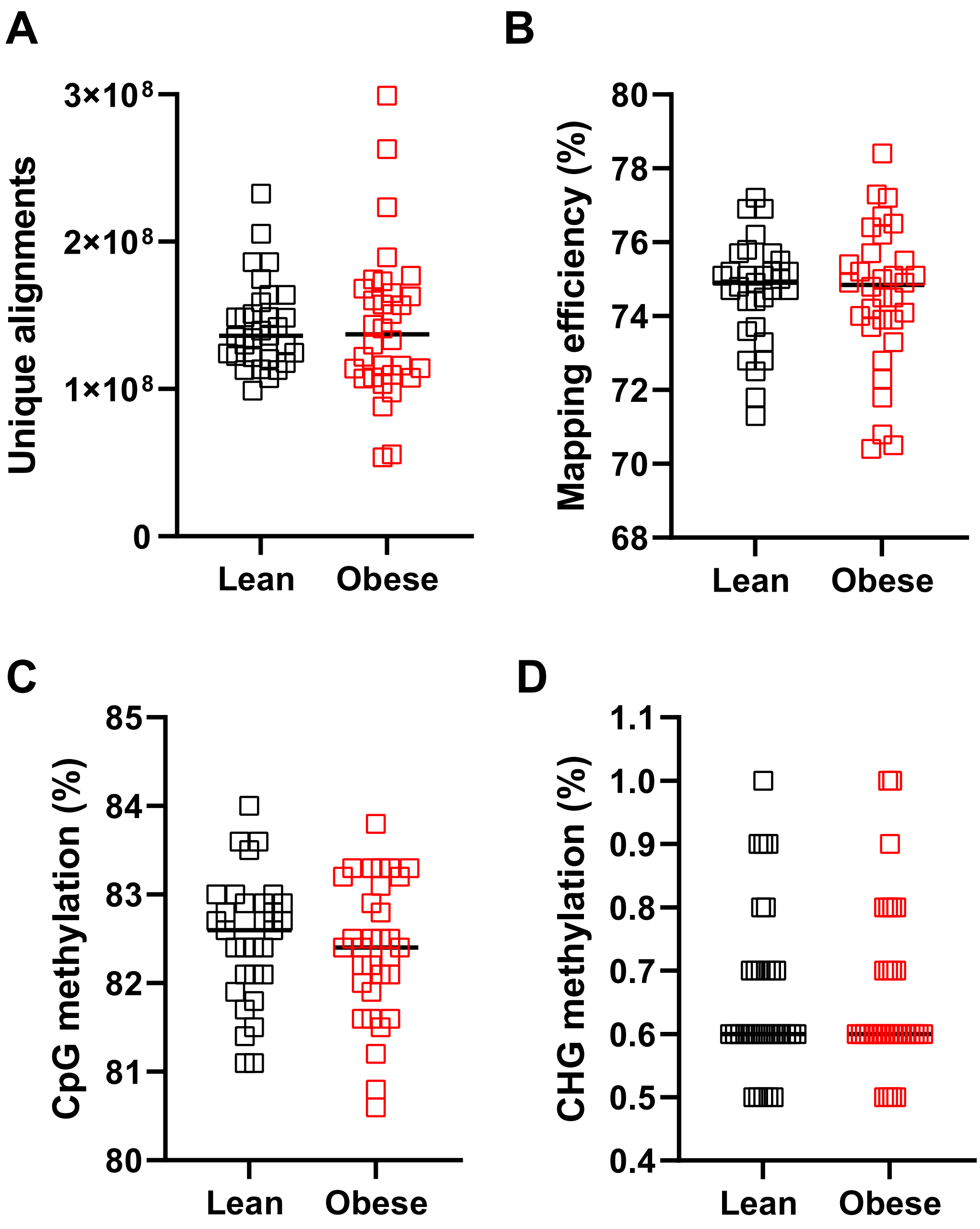

Figure S4. Pairwise comparison of sequencing statistics from the age-matched, multi-ethnic discovery cohort after exclusions based on data quality control. A) Reads uniquely mapped to the whole genome + rDNA consensus are not different between lean or obese males (p = 0.6671, two-sided Mann-Whitney test). **B)** Mapping efficiency is not variable between lean or obese males (p = 0.8031, Mann-Whitney test). **C)** Global CpG methylation estimates are not different between lean or obese males (p = 0.6103, two-sided Mann-Whitney test). **D)** Non-CpG (CHG) methylation is not different between lean or obese males (p = 0.9956, Mann-Whitney test). Throughout lean (BMI<25 kg/m^2, n=31 individual males, black) or obese (BMI>30 kg/m^2, n=32 individual males, red).

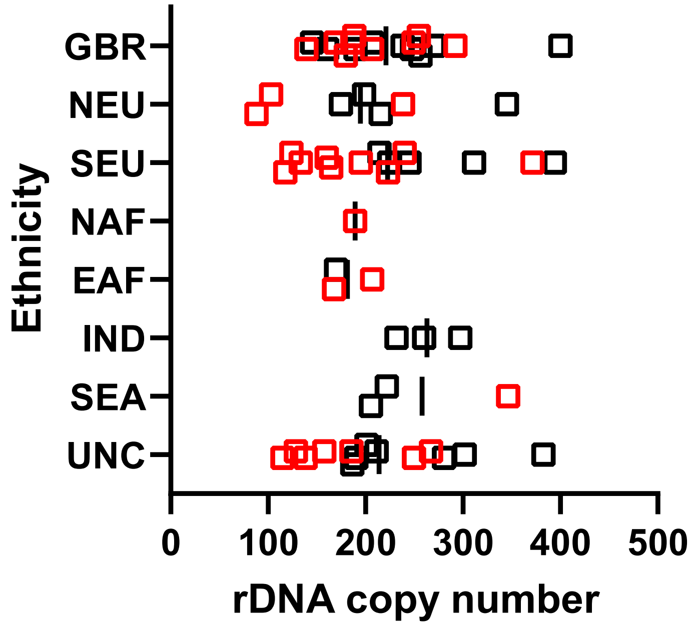

Figure S5. rDNA copy number broken down by ethnicity in the age-matched mixed ethnicity discovery cohort. Throughout lean (BMI<25 kg/m^2, n=31, black individual males) or obese (BMI>30 kg/m^2, n=32, red individual males). GBR (White British), NEU (Northern European), SEU (Southern European), NAF (North African), EAF (East African), IND (Indian), SEA (South East Asian), UNC (unclassified). The trend for lower rDNA copy number reaches significance in SEU (p=0.0420, Mann-Whitney test) and UNC (p=0.0379, two-sided Mann-Whitney test).

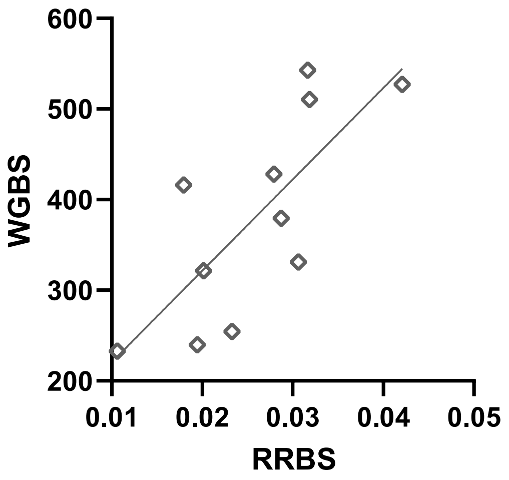

Figure S6. Cross-validation of copy-number estimation methods. WGBS data and RRBS data generated from human LCLs analysed with the respective copy number estimation approaches for each data type show a positive correlation (Spearman r = 0.7727, p = 0.0074, n = 11 individual donors).

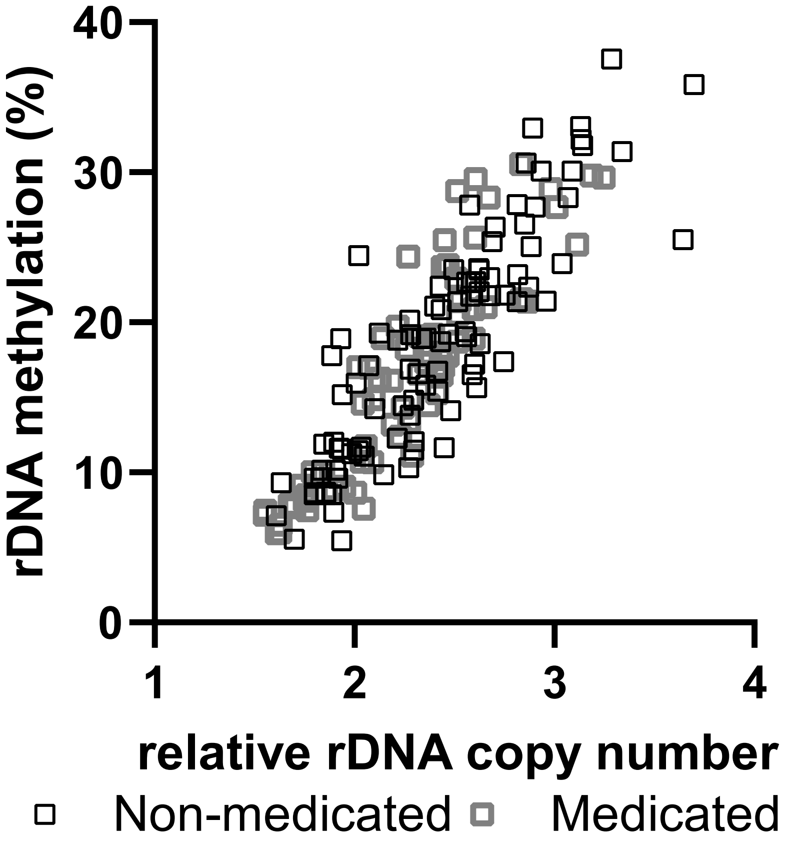

Figure S7. rDNA copy number and methylation are highly correlated in the adipose tissue from the validation (METSIM) cohort. All samples (Spearman r=0.8830, p<0.0001, n=169 individual males). Non-medicated only (Spearman r=0.8803, p<0.0001, n=100 individual males). Medicated only (Spearman r=0.9059, p<0.0001, n=69 individual males).

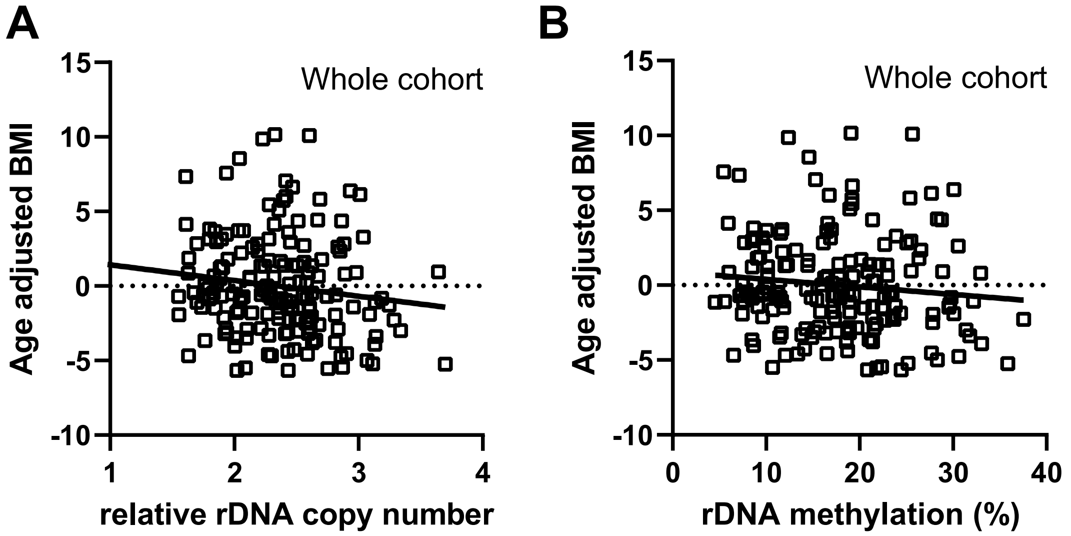

Figure S8. rDNA copy number negatively correlates with age-adjusted BMI but methylation does not. (A) rDNA copy number (Spearman r=-0.1657, p=0.0313, n=169 individual males) **(B)** rDNA methylation (Spearman r=-0.1238, p=0.1087, n=169 individual males).

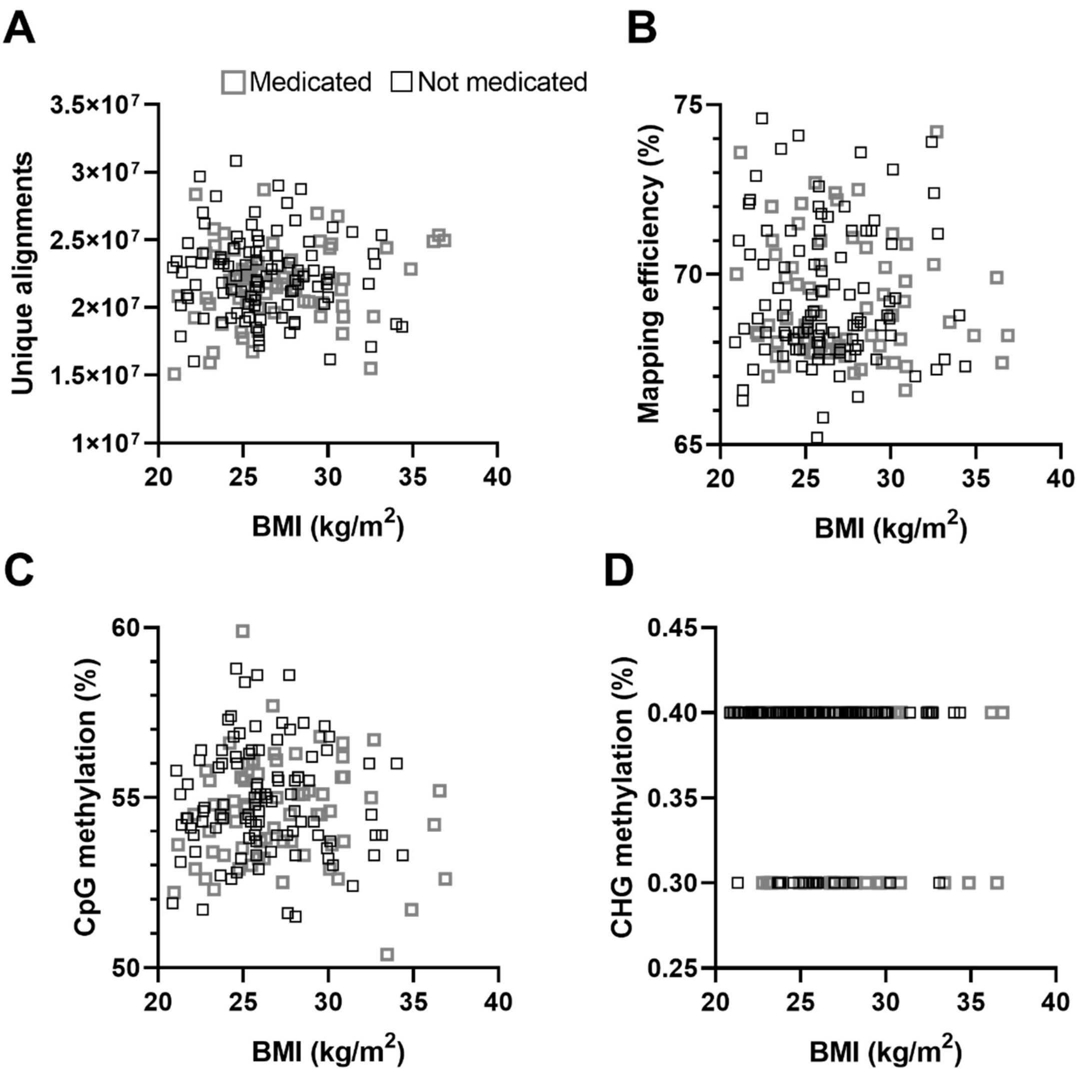

Figure S9. BMI-association for sequencing statistics for the adipose tissue from the METSIM cohort after exclusions based on data quality control. Medicated individuals are represented in grey, individuals not taking any medication are represented in black. **A)** Reads uniquely mapped to the whole genome + rDNA consensus are not correlated with BMI (Medicated: Spearman r = 0.1583, p = 0.1940, n = 69 individual males not medicated: Spearman r = -0.06787, p = 0.5022, n = 100 individual males). **B)** Mapping efficiency is not correlated with BMI (Medicated: Spearman r = -0.08227, p = 0.5016, n= 69 individual males, not medicated: Spearman r = -0.01277, p = 0.8997, n = 100 individual males). **C)** Global CpG methylation estimates are not correlated with BMI (Medicated: Spearman r = 0.09548, p = 0.4351, n= 69, not medicated: Spearman r = -0.02857, p = 0.7778, n = 100 individual males). **D)** Non-CpG (CHG) methylation is not correlated with BMI (Medicated: Spearman r = -0.1065, p = 0.3837, n= 69 individual males, not medicated: Spearman r = 0.02075, p = 0.8376, n = 100 individual males). All tests are two-sided.

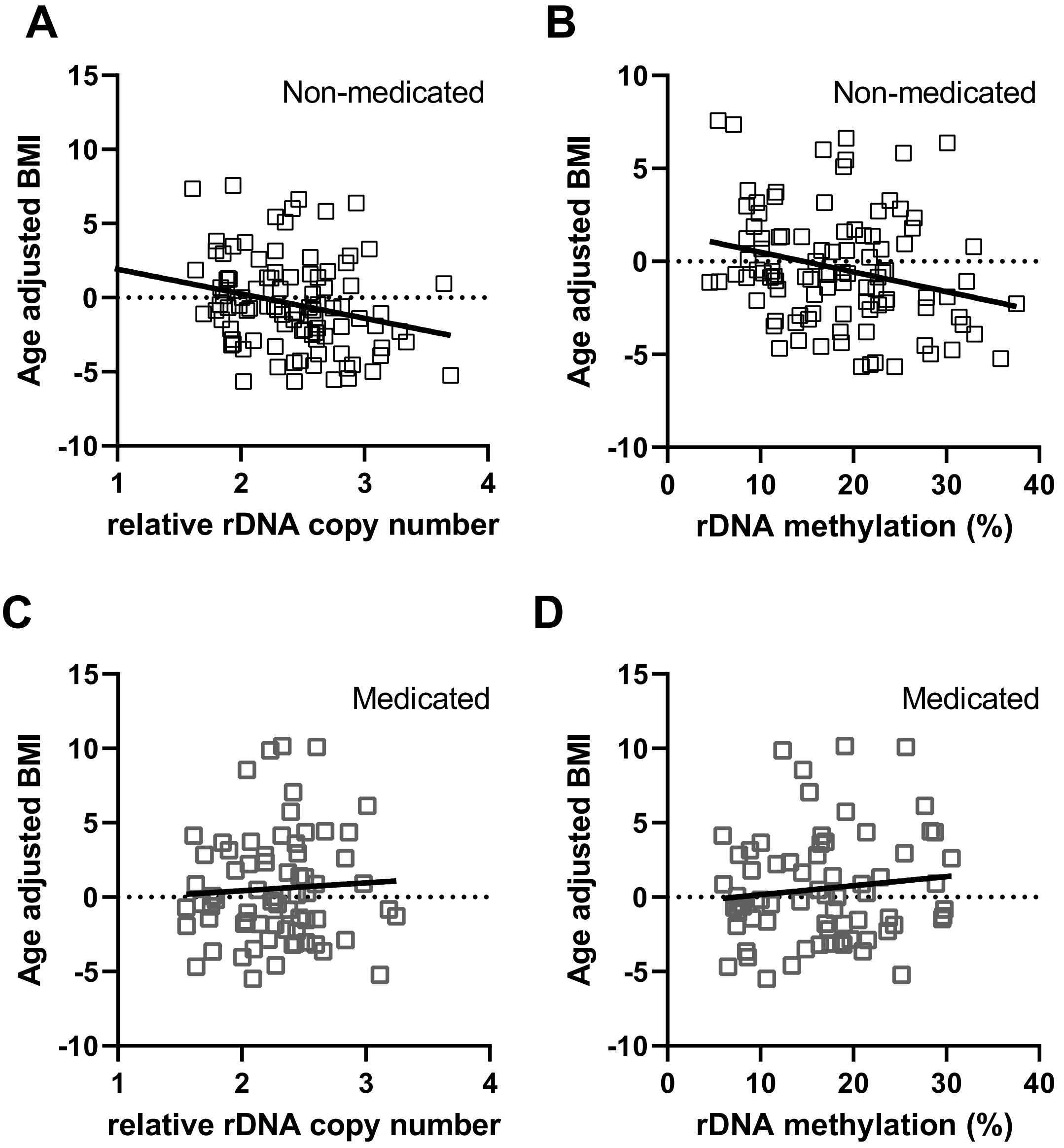

Figure S10. rDNA copy number and methylation correlate with age-adjusted BMI only in the non-medicated group. (A) Relative rDNA copy number is negatively correlated with age-adjusted BMI in the non-medicated group (Spearman r=-0.2865, p=0.0039, n=100 individual males). **(B)** rDNA methylation is negatively correlated with age-adjusted BMI in the non-medicated group (Spearman r=-0.2487, p=0.0126, n=100 individual males). **(C)** Relative rDNA copy number is not correlated with age-adjusted BMI in the non-medicated group (Spearman r=0.0619, p=0.6132, n=69 individual males). **(D)** rDNA methylation is not correlated with age-adjusted BMI in the non-medicated group (Spearman r=-0.0832, p=0.4968, n=69 individual males). All tests are two-sided.

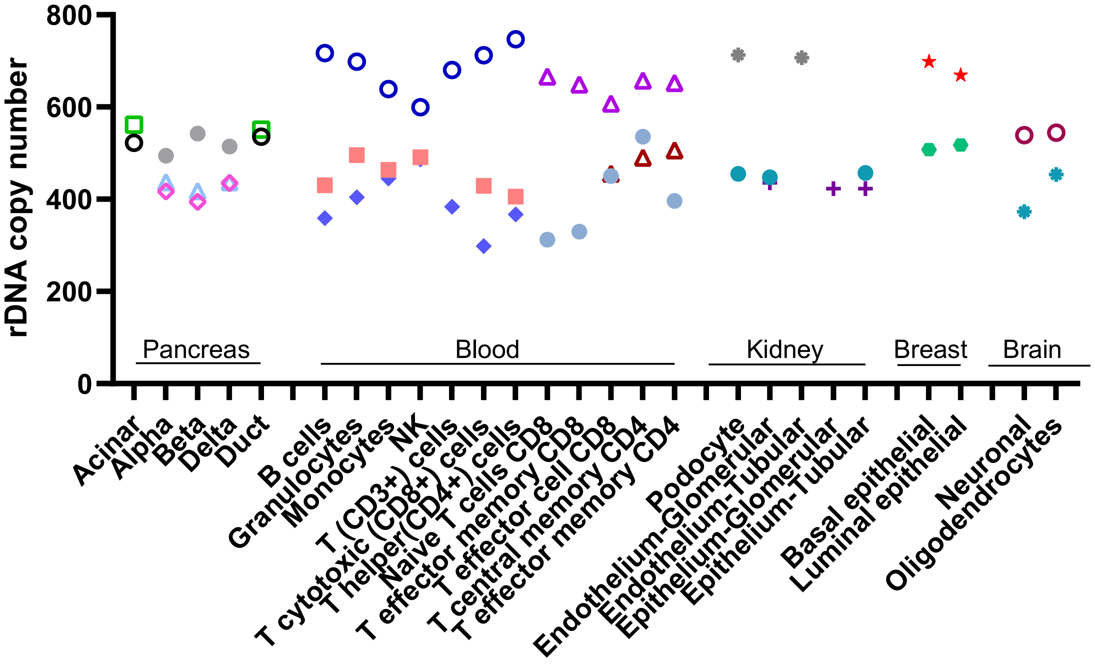

Figure S11. rDNA CN in purified cell populations. Different donors are indicated by the different colour/symbol combinations. Type II ANOVA shows that when all cell types (n=26) are considered, only donor origin influences rDNA CN (p_{donor} = 3.8x10^{-11}, p_{cell_type} = 0.8604). This is also true when only isolated blood cell types are included (p_{donor} = 7.5x10^{-8}, p_{cell_type} = 0.8414).

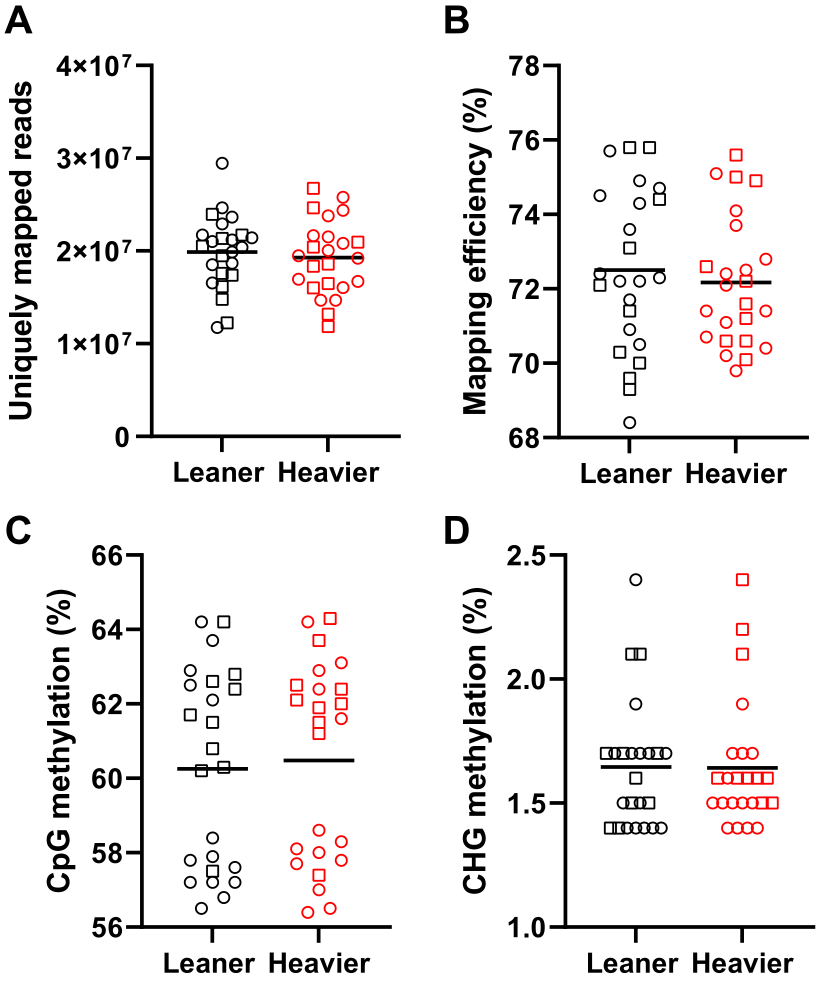

Figure S12. Pairwise comparison of sequencing statistics for single ethnicity, monozygotic twins discordant for BMI after exclusions based on data quality control. A) Reads uniquely mapped to the whole genome + rDNA consensus are not different between leaner or heavier twins (p=0.2182, n=24 twin pairs, two-sided Wilcoxon matched-pairs signed rank test, Pairing: Spearman r=0.4843, p=0.0082). **B)** Mapping efficiency is not variable between leaner or heavier twins (p=0.2101, n=24 twin pairs, two-sided Wilcoxon matched-pairs signed rank test, Pairing: Spearman r=0.5727, p=0.0017). **C)** Global CpG methylation estimates are not different between leaner or heavier twins (p=0.2311, n=24 twin pairs, two-sided Wilcoxon matched-pairs signed rank test, Pairing: Spearman r=0.8520, p<0.0001). **D)** Non-CpG (CHG) methylation is not different between leaner or heavier twins (p=0.9414, n=24 twin pairs, two-sided Wilcoxon matched-pairs signed rank test, Pairing: Spearman r=0.5859, p=0.0013). Sex of twins is indicated (Male=square, Female=circle).

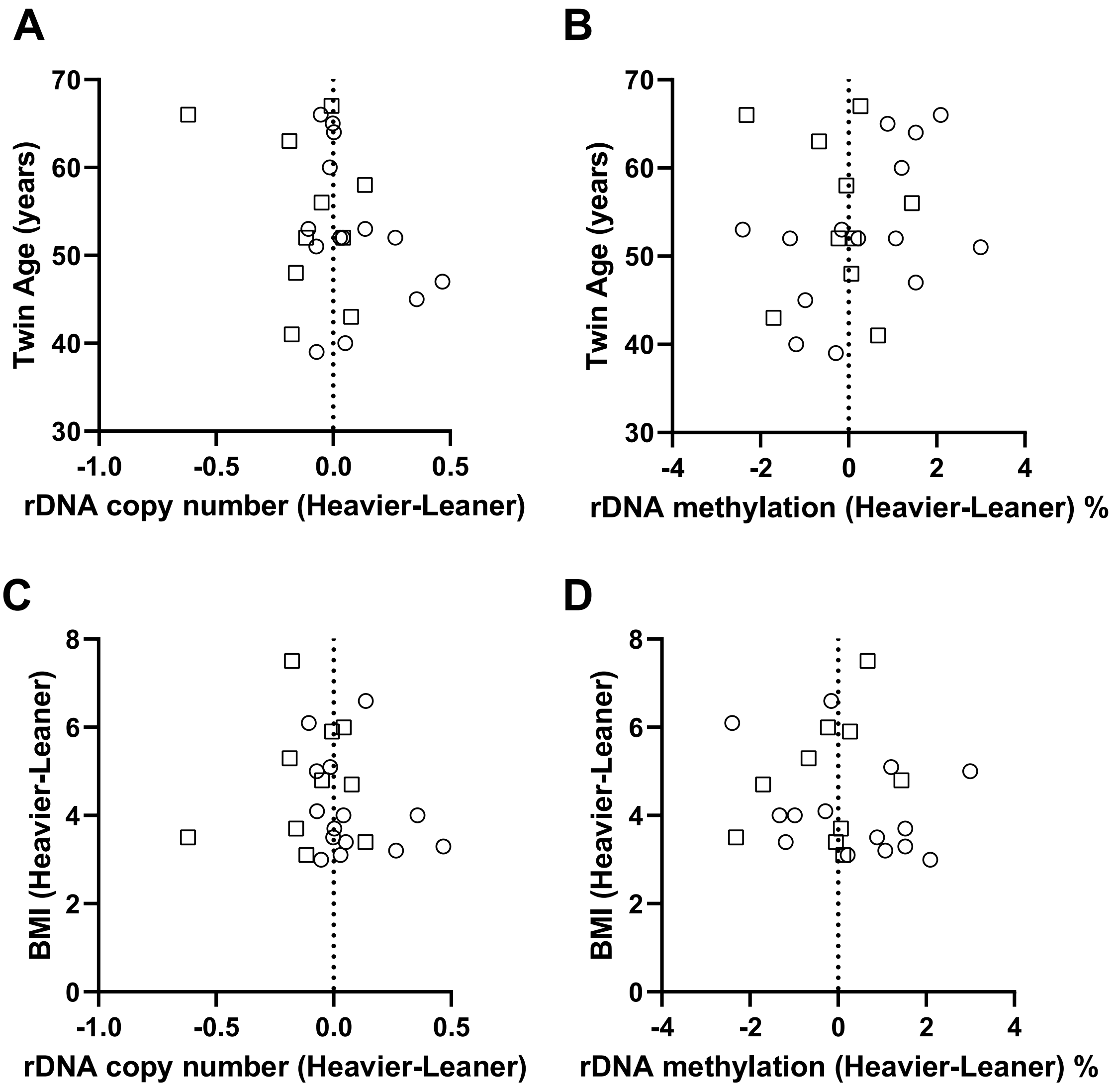

Figure S13. Age and BMI discordance are not associated with the magnitude of between-twin differences in rDNA copy number or methylation. Female twin pairs are indicated by circles, male twin pairs by squares. **(A)** There is no correlation between the age of twin pairs and the between-twin difference in estimated relative copy number (Spearman r = -0.2267, p = 0.2867, n = 24). **(B)** There is no correlation between the age of twin pairs and the between-twin difference in rDNA methylation (Spearman r = 0.2141, p = 0.3152, n = 24). **(C)** There is no correlation between the BMI discordance of twin pairs and the between-twin difference in estimated relative copy number (Spearman r = -0.2128, p = 0.3180, n = 23). **(D)** There is no correlation between the BMI discordance of twin pairs and the between-twin difference in rDNA methylation (Spearman r = -0.2224, p = 0.2962, n = 24).

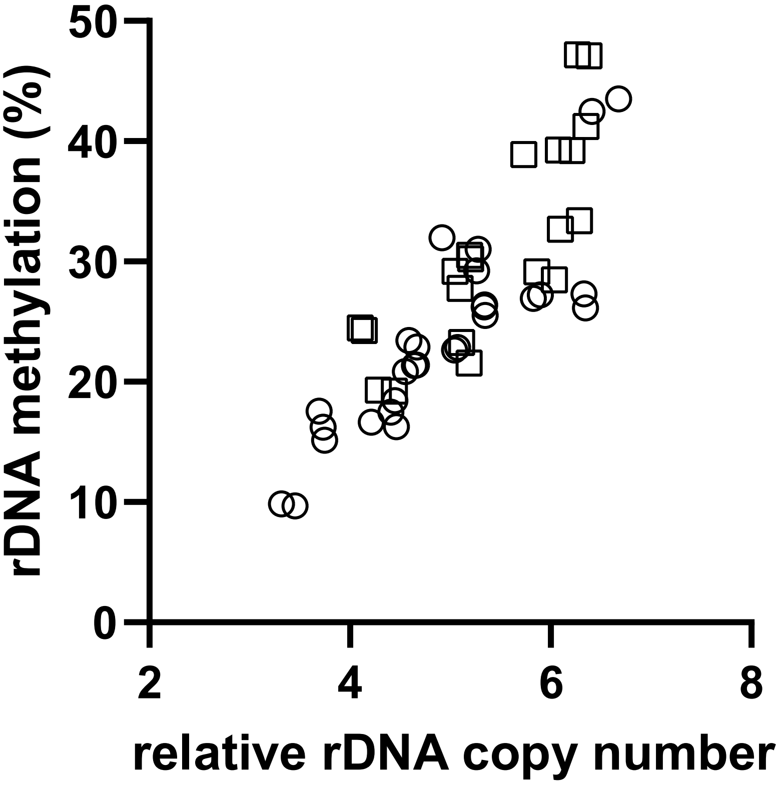

Figure S14. rDNA copy number and methylation are highly correlated in the blood from the single ethnicity monozygotic twin cohort. Males are indicated by squares, females by circles (two-sided Spearman r=0.8564, p<0.0001, n=48 individuals in total).

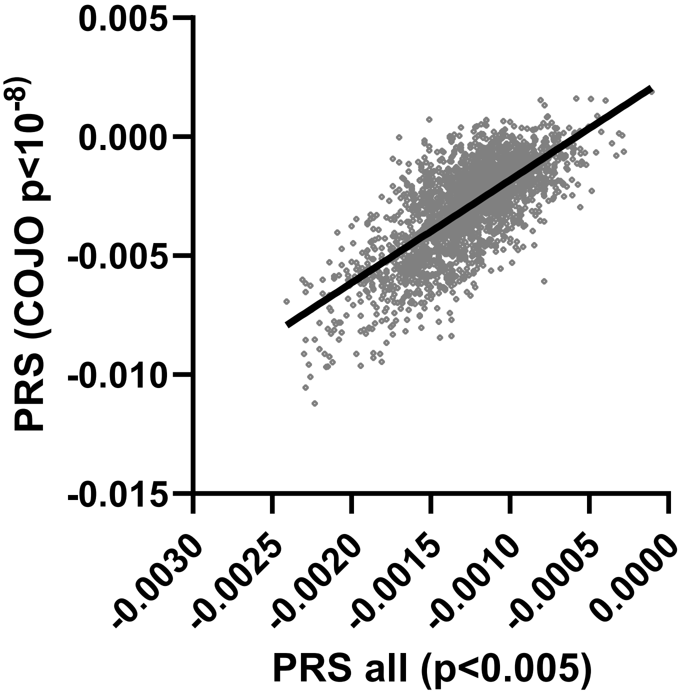

Figure S15. Correlation of the polygenic risk scores for BMI in the 1000 Genomes Project samples calculated using SNVs that produce the best model for explaining rDNA variance (no COJO, P<0.005) or the COJO filtered SNVs previously used[1]. Spearman r=0.6734, p<0.0001, n=2390 individuals.

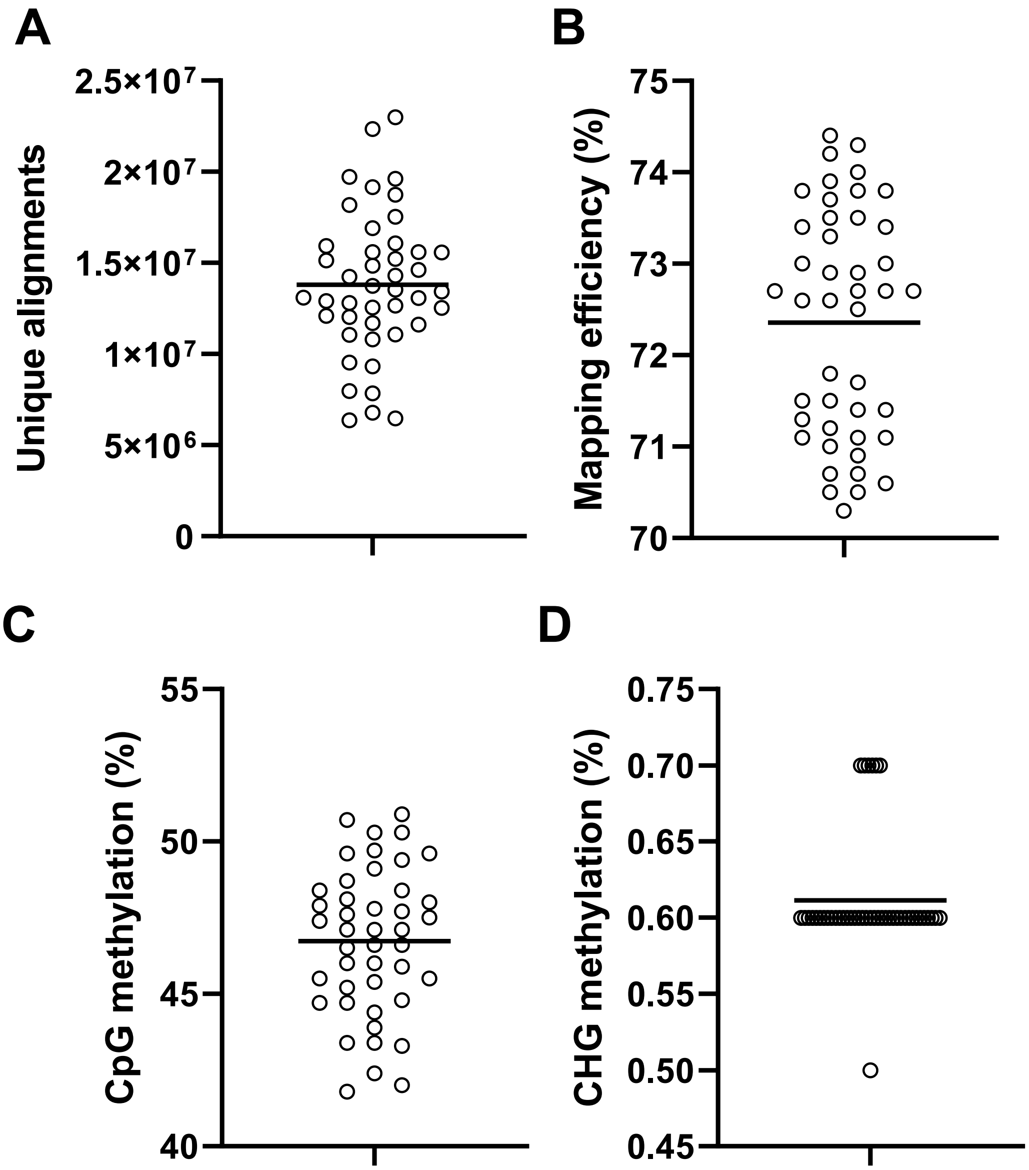

Figure S16. Distribution of sequencing statistics for RRBS data from liver of Sprague-Dawley rats after exclusions based on data quality control. **A)** The distribution of reads uniquely mapped to the whole genome + rDNA consensus are shown. **B)** Mapping efficiency distribution is shown **C)** Global CpG methylation estimate distribution is shown. **D)** Non-CpG (CHG) methylation distribution is shown.

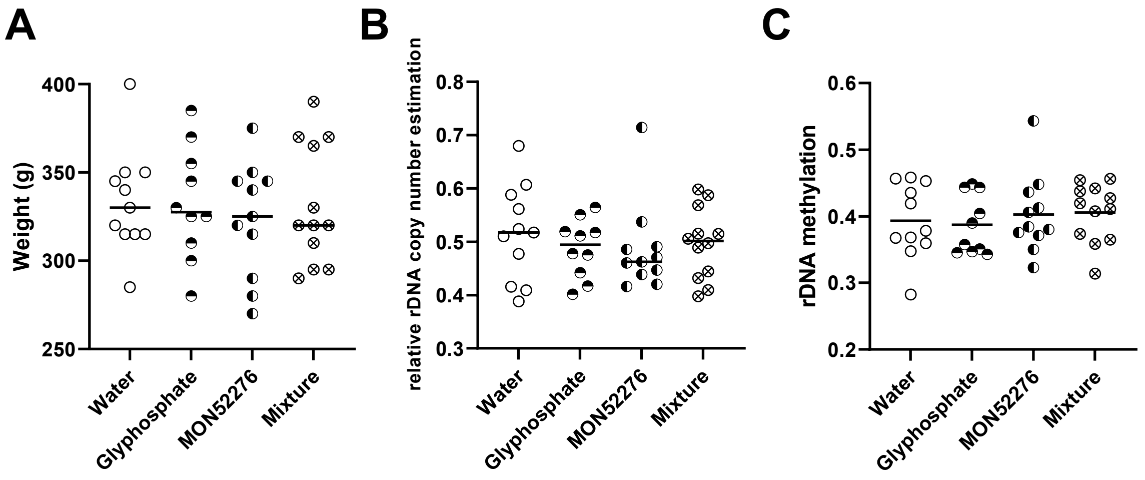

Figure S17. Chemical treatments administered to female Sprague Dawley rats in the original study do not associate with changes in body mass, rDNA copy number or methylation. (A) The body mass at the end of the treatment period is not altered by any treatment compared to water (Kruskal-Wallis test, p=0.9352). **(B)** The rDNA copy number is not changes in any treatment compared to water (Kruskal-Wallis test, p=0.7205). **(C)** The rDNA methylation is not changes in any treatment compared to water (Kruskal-Wallis test, p=0.7231).

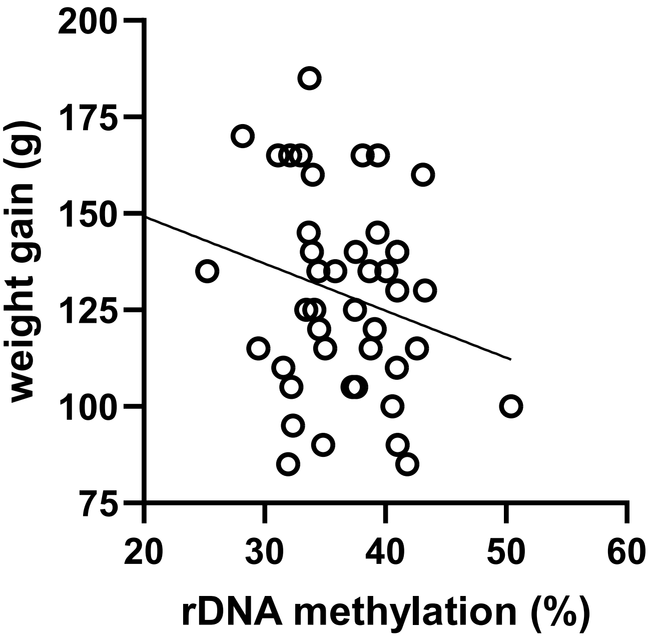

Figure S18. The weight gained throughout the study period is not significantly correlated with methylation at rDNA. Two-sided Spearman r=-0.2570, p=0.0922, n=44 individual rats.

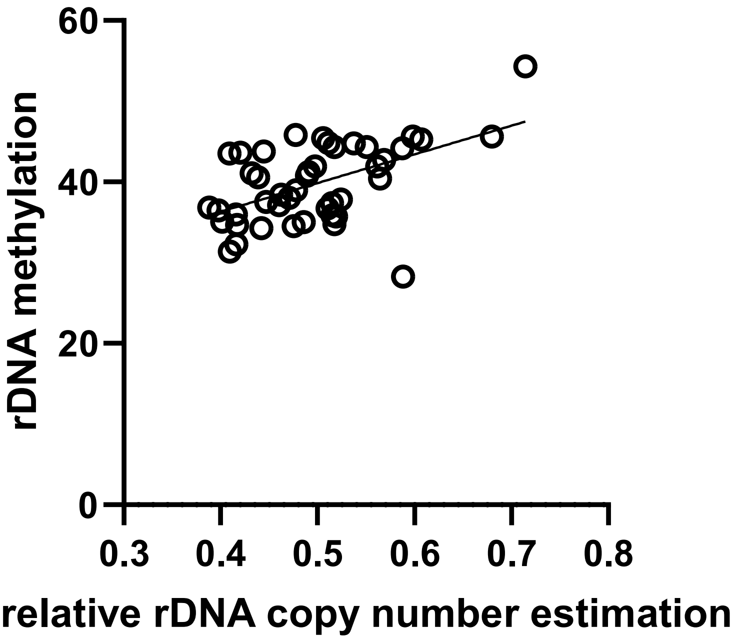

Figure S19. rDNA copy number and methylation are correlated in the liver of Sprague Dawley rats. Two-sided Spearman r=0.4620, p=0.0016, n=44 individual rats.

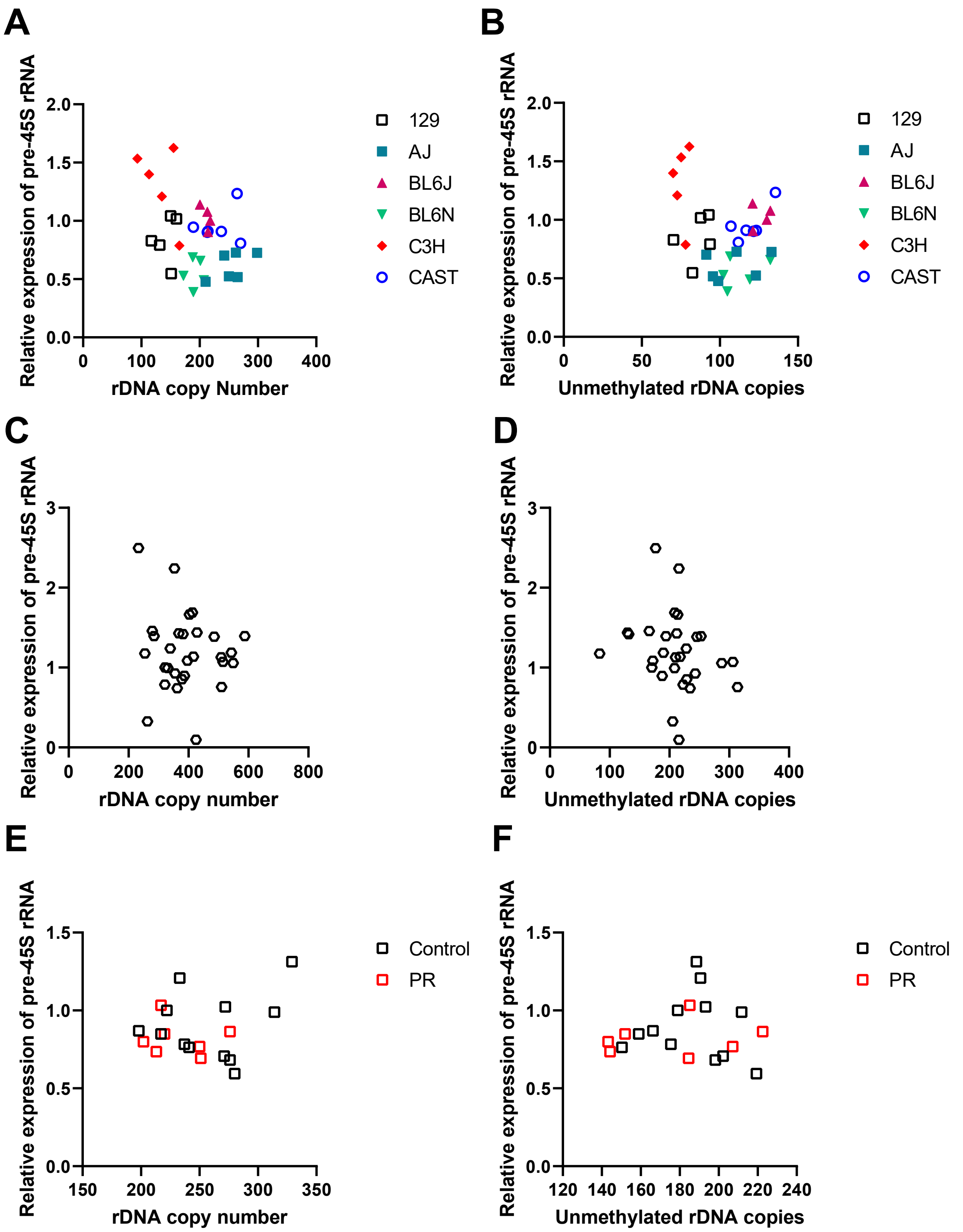

Figure S20. Nascent 45S-rRNA transcript abundance does not correlate with total rDNA copy number or the number of unmethylated copies present in adult tissues. Kidney tissue across different mouse strains **(A)** Spearman r=-0.3074, p=0.0926, n=31 individual mice **(B)** Spearman r=-0.1266, p=0.4973, n=31 individual mice. Human LCLCs **(C)** Spearman r=-0.04680, p=0.8095, n=29 individual donors, **(D)** Spearman r=-0.3340, p=0.0766, n=29 individual donors. From liver of C57BL/6J mice **(E)** Spearman r=-0.08827, p=0.7193, n=19 individual mice **(F)** Spearman r=-0.0009, p=0.9975, n=19 individual mice.

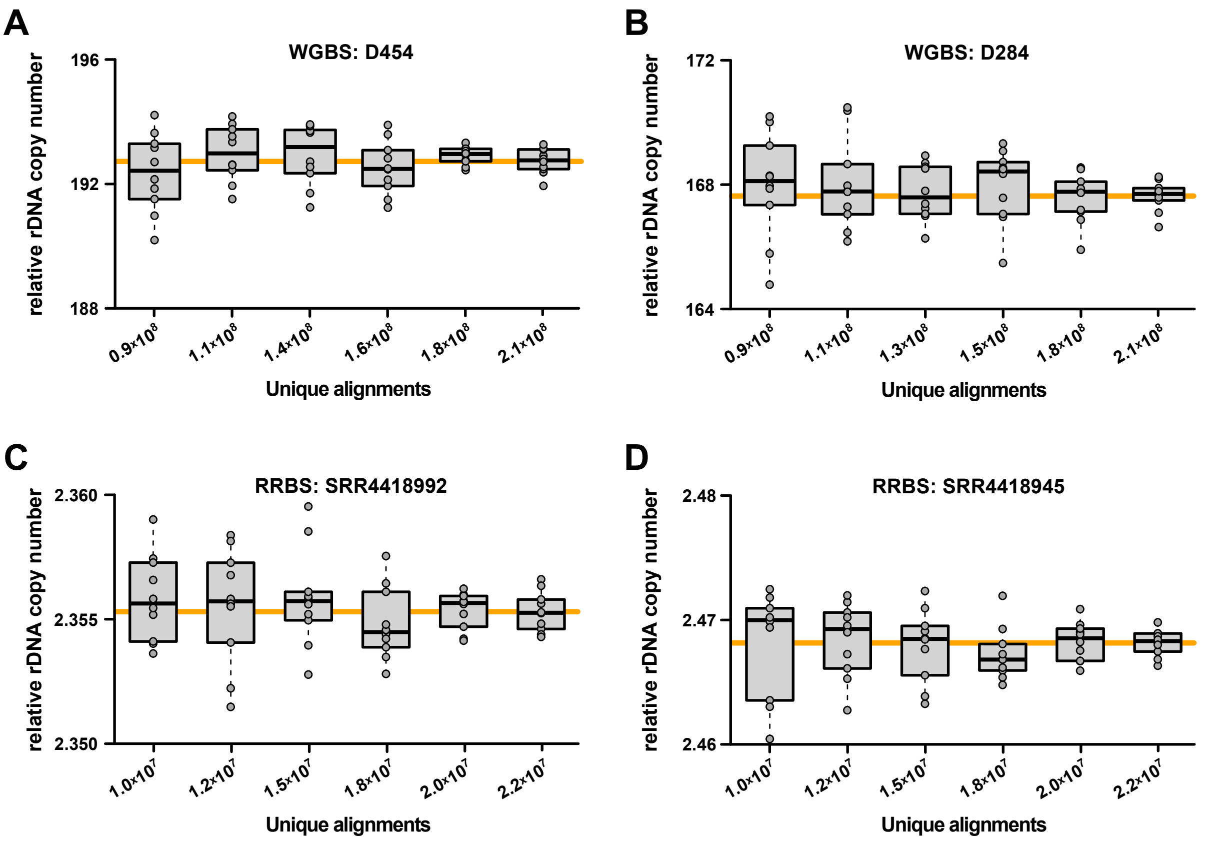

Figure S21. Read coverage downsampling affects rDNA copy number estimate variability in WGBS and RRBS datasets. High-coverage WGBS (A, B) and RRBS (C, D) samples are selected for downsampling at levels of 40%, 50%, 60%, 70%, 80% and 90%. At each downsampling level, 10 random downsamplings were performed. Decreasing read coverage increases the deviation of rDNA copy number estimates while the mean stays close to the value estimated in the full dataset (orange line).

Table S1. Summary metabolic data for the lean and obese male cohort. Indicators of Type 2 Diabetes are from collected data and defined according to the recommendations of the World Health Organisation of HbA1c(%)> 6.5 for Diabetic and 6<HbA1c(%)<6.5 for pre-diabetic [2]. S.D= standard deviation.

**Trait**	**Lean**	**Obese**	**Count**	**Two-sided Mann-Whitney test**
	Mean ± S.D.	Mean ± S.D	(lean, obese)	P value
BMI (kg/m^2)	22.37 ± 1.73	33.77 ± 3.06	31,32	P = 9.5 x10^{-12}
Age (years)	35.66 ± 5.27	37.38 ± 5.07	31,32	P = 0.1154
Waist circumference (cm)	81.85 ± 6.66	111.72 ± 9.36	31,32	P = 1.2 x10^{-11}
Systolic BP (mmHg)	118.5 ± 10.90	133.17 ± 11.28	31,32	P = 3.5 x10^{-6}
Diastolic BP (mm/Hg)	75.74 ± 8.46	83.55 ± 8.72	31,32	P = 0.0004
C-reactive protein (mg/L)	1.28 ± 1.94	2.40 ± 2.69	31,31	P = 0.0006
Fasting Glucose (mmol/L)	4.66 ± 0.37	4.96 ± 0.43	31,32	P = 0.0054
HbA1c (%)	5.03 ± 0.98	5.38 ± 0.29	31,31	P = 0.0214
Fasting insulin (mIU/L)	4.85 ± 3.39	12.20 ± 7.96	31,31	P = 5.8 x10^{-6}
HOMA-IR	1.02 ± 0.77	2.74 ± 1.95	31,31	P = 8.0 x10^{-6}
Total cholesterol (mmol/L)	4.77 ± 0.97	5.15 ± 0.95	31,32	P = 0.1044
Triglycerides (mmol/L)	1.09 ± 0.59	1.67 ± 0.93	31,31	P = 0.0034
HDL (mmol/L)	1.47 ± 0.29	1.23 ± 0.24	31,32	P = 0.0013
LDL (mmol/L)	2.85 ± 0.90	3.17 ± 0.77	31,32	P = 0.1248
Cholesterol:HDL	3.36 ± 0.94	4.10 ± 1.23	31,32	P = 0.0026
Self-identified ever smoker	8	6	31,31	
Indicators of Type 2 Diabetes	0	0	31,31	

Table S2. Mean coverage of genomic features included in methylation analyses. Values presented are average reads/base in each feature group. rDNA values represent the entire rDNA unit, inclusive of the intergenic spacer.

	Lean		Obese		**Two-sided Mann-Whitney test**
	Mean	S.D.	Mean	S.D.	P value
rDNA	109.57	39.89	100.91	80.20	0.0622
Promoters	302.61	74.60	313.08	154.88	0.5075
Exons	41.48	10.00	42.49	18.37	0.5524
Introns	471.24	123.11	478.60	191.47	0.7796
CpG Islands	229.45	56.41	238.66	124.43	0.4564
CpG shores	200.75	47.71	208.90	100.54	0.6181
Simple Repeats	225.58	68.67	219.46	62.77	0.6972
tRNA	31.11	7.78	32.07	14.00	0.6014
LINE	29.55	8.40	30.04	10.94	0.9103
LTR	23.20	5.99	23.19	9.02	0.6181
Retroposon	95.02	19.17	97.69	43.66	0.4816
Satellite	458.42	134.56	431.55	140.06	0.4319
SINE	34.11	8.90	35.05	14.51	0.7979

Table S3. Ethnic breakdown of lean and obese groups within mixed-ethnicity cohort

Ethnicity	Lean	Obese
White British (GBR)	9	9
Northern European (NEU)	4	3
Southern European (SEU)	5	9
North African (NAF)	0	1
East African (EAF)	1	2
Indian (IND)	3	0
South East Asian (SEA)	2	1
Unclassified (UNC)	7	7
Total	31	32

Table S4. Summary phenotype data for included individuals from the METSIM cohort. Summary statistics for individuals classified by whether they take regular medications or not. Indicators of Type 2 Diabetes are from collected data or previous clinical diagnosis and defined according to the recommendations of the World Health Organisation [2]. S.D= standard deviation. Comparisons that pass the nominal threshold of P < 0.05 are indicated in bold.

Trait	Medicated n=69	Not medicated n=100	Two sided Mann Whitney test
	Mean ± S.D.	Mean ± S.D.	P value
Lean (BMI<25 kg/m^2)	31.88%	35.00%	
Overweight (25<BMI<30 kg/m^2)	44.93%	53.00%	
Obese (BMI>30 kg/m^2)	23.19%	12.00%	
BMI at recruitment (kg/m^2)	27.16 ± 3.68	26.22 ± 3.11	P = 0.1488
BMI at biopsy (kg/m^2)	27.16 ± 3.76	26.14 ± 2.99	P = 0.2207
Age (years)	55.03 ± 3.71	54.34 ± 4.65	P = 0.2387
Weight (kg)	84.26 ± 12.78	81.59 ± 10.60	P = 0.2989
Height (cm)	176.0 ± 5.43	176.2 ± 6.02	P = 0.6636
Waist circumference (cm)	98.78 ± 10.58	95.00 ± 9.20	**P = 0.0396**
Hip circumference (cm)	101.1 ± 7.26	100.0 ± 5.16	P = 0.3205
Waist to Hip ratio	0.98 ± 0.06	0.95 ± 0.06	**P = 0.0046**
Systolic BP (mmHg)	138.9 ± 17.31	133.4 ± 12.31	P = 0.0926
Diastolic BP (mm/Hg)	90.31 ± 9.93	86.97 ± 7.55	**P = 0.0480**
C-reactive protein (mg/L)	2.55 ± 5.33	2.02 ± 2.53	P = 0.5438
Fasting glucose recruitment (mmol/L)	5.80 ± 0.50	5.76 ± 0.48	P = 0.7147
Fasting glucose biopsy (mmol/L)	5.57 ± 0.55	5.50 ± 0.44	P = 0.6698
HbA1c (%)	5.65 ± 0.33	5.62 ± 0.30	P = 0.4598
Fasting insulin recruitment (mIU/L)	8.48 ± 4.66	6.66 ± 3.57	**P = 0.0130**
HOMA-IR	2.22 ± 1.27	1.72 ± 0.99	**P = 0.0163**
Total cholesterol (mmol/L)	5.46 ± 0.82	5.64 ± 0.87	P = 0.2102
Triglycerides (mmol/L)	1.45 ± 0.74	1.34 ± 0.95	P = 0.0527
HDL (mmol/L)	1.48 ± 0.36	1.54 ± 0.43	P = 0.3180
LDL(mmol/L)	3.42 ± 0.75	3.56 ± 0.72	P = 0.1873
Cholesterol:HDL	3.88 ± 0.99	3.90 ± 1.05	P = 0.9358
Self-identified ever smoker	62.32%	51.00%	
T2D: Previously diagnosed or HbA1c (%) ≥ 6.5	7.25 %	4.00%	
Prediabetic: 6 ≤ Hb1Ac (%) < 6.5	17.38%	12:00%	

Table S5. rDNA copy number and methylation correlation statistics with phenotypes in the mixed ethnicity cohort and the unmedicated METSIM cohort. Phenotypic variables available for the mixed-ethnicity obese male cohort and the unmedicated METSIM (Finnish) male cohort were correlated with rDNA copy number (top panel) and rDNA methylation (lower panel). Correlations with a P value <0.05 are in bold. Only individuals with data for all variables were included.

	Mixed ethnicity cohort (n=63)		METSIM cohort (n=100)	
	Correlation with rDNA copy number		Correlation with rDNA copy number	
Trait	Two-sided Spearman r	P value	Two-sided Spearman r	P value
BMI (kg/m^2)	**-0.3655**	**0.0035**	**-0.2992**	**0.0025**
Age (years)	-0.0705	0.5860	0.1636	0.1039
Waist circumference (cm)	**-0.3607**	**0.0040**	**-0.2487**	**0.0126**
Systolic BP (mmHg)	-0.1307	0.3113	**-0.2023**	**0.0436**
Diastolic BP (mm/Hg)	-0.1444	0.2628	**-0.2539**	**0.0109**
C-reactive protein (mg/L)	**-0.4614**	**0.0002**	-0.1153	0.2531
Fasting Glucose (mmol/L)	-0.0936	0.4691	-0.0691	0.4948
HbA1c (%)	-0.2285	0.0741	0.0110	0.9135
Fasting insulin (mIU/L)	**-0.3110**	**0.0139**	-0.1652	0.1004
HOMA-IR	**-0.3001**	**0.0178**	-0.1684	0.0941
Total cholesterol (mmol/L)	-0.0060	0.9629	-0.1459	0.1457
Triglycerides (mmol/L)	-0.0305	0.8142	-0.0273	0.7878
HDL (mmol/L)	0.2482	0.0517	-0.1168	0.2470
LDL(mmol/L)	-0.0777	0.5484	-0.1214	0.2287
Cholesterol:HDL	-0.0758	0.5582	0.0686	0.4979
	Mixed ethnicity cohort (n=63)		METSIM cohort (n=100)	
	Correlation with rDNA methylation		Correlation with rDNA methylation	
Trait	Two-sided Spearman r	P value	Two-sided Spearman r	P value
BMI (kg/m^2)	-0.2352	0.0658	**-0.2518**	**0.0115**
Age (years)	-0.0022	0.9862	0.0545	0.5903
Waist circumference (cm)	-0.2261	0.0772	**-0.2080**	**0.0378**
Systolic BP (mmHg)	-0.1689	0.1894	**-0.2245**	**0.0247**
Diastolic BP (mm/Hg)	-0.1501	0.2442	-0.1791	0.0746
C-reactive protein (mg/L)	**-0.4344**	**0.0004**	-0.1507	0.1344
Fasting Glucose (mmol/L)	-0.1880	0.1434	0.0243	0.8101
HbA1c(%)	-0.2077	0.1053	0.1004	0.3203
Fasting insulin (mIU/L)	**-0.2559**	**0.0447**	-0.1223	0.2255
HOMA-IR	-0.2460	0.0539	-0.1172	0.2453
Total cholesterol (mmol/L)	-0.0074	0.9544	-0.1254	0.2137
Triglycerides (mmol/L)	0.0068	0.9582	-0.0613	0.5450
HDL (mmol/L)	0.1552	0.2283	-0.0639	0.5276
LDL(mmol/L)	-0.0721	0.5777	-0.1082	0.2838
Cholesterol:HDL	-0.0608	0.6389	0.0064	0.9493

Table S6. Summary data of cohort characteristics for monozygotic twin data.

			Leaner	Heavier	
	Number of MZ twin pairs	Range of BMI discordance (kg/m^2)	Average BMI ± S.D (kg/m^2)	Average BMI ± S.D (kg/m^2)	Average Age ± S.D (years)
Female	14	3-6.6	22.84 ± 3.44	27.99 ± 4.15	52.8 ± 8.6
Male	10	3.1-7.5	21.76 ± 2.54	26.55 ± 2.43	54.6 ± 9.1

Table S7. Correlation between weight at each time point with rDNA copy number in the liver of female Sprague Dawley rats. n=44 at each time point. Measurement with P<0.05 are indicated in bold.

Measurement week	Two-sided Spearman r	P value
8	-0.0718	0.6432
9	-0.1579	0.3088
10	-0.1990	0.1953
11	-0.2875	0.0585
12	-0.2119	0.1673
13	-0.1972	0.1995
14	-0.2689	0.0775
15	-0.2776	0.0681
16	-0.2294	0.1341
17	-0.2788	0.0669
**18**	**-0.3294**	**0.0290**
**19**	**-0.3652**	**0.0148**

Table S8. Masked regions in the human reference sequence

Assembly	Contig	Start	End
Hg38	Chr 1	91387225	91387553
Hg38	Chr 21	8202092	8260970
Hg38	Chr 21	8385101	8472093
Hg38	Chr 21	8986700	9899746
Hg38	ChrUn_GL000220v1	0	161802
Hg38	Chr22_KI270733v1_random	0	179772
Rn7	Chr 3	2231807	2240483
Rn7	chrUn_NW_023637849v1	61191	69866
Rn7	chrUn_NW_023637831v1	81687	90364

Supplementary References

1. Yengo, L., Sidorenko, J., Kemper, K.E., Zheng, Z., Wood, A.R., Weedon, M.N., Frayling, T.M., Hirschhorn, J., Yang, J., Visscher, P.M., and Consortium, G. (2018). Meta-analysis of genome-wide association studies for height and body mass index in approximately 700000 individuals of European ancestry. Hum Mol Genet *27*, 3641-3649. 10.1093/hmg/ddy271.
2. (2011). In Use of Glycated Haemoglobin (HbA1c) in the Diagnosis of Diabetes Mellitus: Abbreviated Report of a WHO Consultation.
