# Supplementary material for: Ribosomal DNA copy number is associated with body mass in humans and other mammals
Source: Nat Commun. 2024 Jun 12;15:5006. doi: 10.1038/s41467-024-49397-5 (PMC11169392; doi:10.1038/s41467-024-49397-5)
Supplement: Supplementary file 1 [file 41467_2024_49397_MOESM3_ESM.pdf]

Description of Additional Supplementary Files

File Name: Supplementary Data 1
Description: Whole genome bisulfite sequencing quality control from blood for mixed ethnicity, age-matched lean and obese males (discovery cohort).

File Name: Supplementary Data 2
Description: Reduced representation bisulfite sequencing quality control for adipose tissue from the METSIM cohort.

File Name: Supplementary Data 3
Description: Reduced representation bisulfite sequencing quality control from blood for single ethnicity, mixed sex monozygotic twin cohort.

File Name: Supplementary Data 4
Description: Reduced representation bisulfite sequencing quality control from liver of Sprague-Dawley rats.

File Name: Supplementary Data 5
Description: Digital PCR rDNA CN and Lambda values for the discovery cohort.
