# Supplementary material for: Ribosomal DNA copy number is associated with body mass in humans and other mammals
Source: Nat Commun. 2024 Jun 12;15:5006. doi: 10.1038/s41467-024-49397-5 (PMC11169392; doi:10.1038/s41467-024-49397-5)
Supplement: Supplementary file 2 [file 41467_2024_49397_MOESM9_ESM.pdf]

nature portfolio

Corresponding author(s): Michelle L Holland

Last updated by author(s): 25/04/2024

Reporting Summary

Nature Portfolio wishes to improve the reproducibility of the work that we publish. This form provides structure for consistency and transparency in reporting. For further information on Nature Portfolio policies, see our Editorial Policies and the Editorial Policy Checklist.

Statistics

For all statistical analyses, confirm that the following items are present in the figure legend, table legend, main text, or Methods section.

n/a	Confirmed	
☐	☒	The exact sample size (*n*) for each experimental group/condition, given as a discrete number and unit of measurement
☐	☒	A statement on whether measurements were taken from distinct samples or whether the same sample was measured repeatedly
☐	☒	The statistical test(s) used AND whether they are one- or two-sided<br>*Only common tests should be described solely by name; describe more complex techniques in the Methods section.*
☐	☒	A description of all covariates tested
☐	☒	A description of any assumptions or corrections, such as tests of normality and adjustment for multiple comparisons
☐	☒	A full description of the statistical parameters including central tendency (e.g. means) or other basic estimates (e.g. regression coefficient) AND variation (e.g. standard deviation) or associated estimates of uncertainty (e.g. confidence intervals)
☐	☒	For null hypothesis testing, the test statistic (e.g. *F*, *t*, *r*) with confidence intervals, effect sizes, degrees of freedom and *P* value noted<br>*Give P values as exact values whenever suitable.*
☒	☐	For Bayesian analysis, information on the choice of priors and Markov chain Monte Carlo settings
☒	☐	For hierarchical and complex designs, identification of the appropriate level for tests and full reporting of outcomes
☒	☐	Estimates of effect sizes (e.g. Cohen's *d*, Pearson's *r*), indicating how they were calculated

Our web collection on statistics for biologists contains articles on many of the points above.

Software and code

Policy information about availability of computer code

Data collection	QuantStudio Absolute Q Digital PCR Software (v.6.3.0) was used for the human digital droplet PCR data collection.
Data analysis	fastqc version 0.11.9, trimgalore version 0.6.5, bismark version 0.23.0, samtools version 1.10, methylKit version 1.16.0, ldsc version 1.0.1, rtracklayer package version 1.54.0, plink2 version 2.0-20200328, blastn version 2.7.1+

For manuscripts utilizing custom algorithms or software that are central to the research but not yet described in published literature, software must be made available to editors and reviewers. We strongly encourage code deposition in a community repository (e.g. GitHub). See the Nature Portfolio guidelines for submitting code & software for further information.

Data

Policy information about availability of data

All manuscripts must include a data availability statement. This statement should provide the following information, where applicable:

- Accession codes, unique identifiers, or web links for publicly available datasets
- A description of any restrictions on data availability
- For clinical datasets or third party data, please ensure that the statement adheres to our policy

Whole genome bisufite sequencing data generated for this study will be available from The Sequence read Archive (Home - SRA - NCBI (nih.gov)) using Bioproject ID: PRJNA817350 upon manuscript acceptance.
External data sources were available in public repositories: GEO (GSE87893), (GSE157551), European Genome Archive (Dataset ID EGAD00001009789) and 1000

Genomes Project. According to the Danish and EU legislations, transfer and sharing of individual-level data require prior approval from the Danish Data Protection Agency and require that data sharing requests are dealt with on a case-by-case basis. For this reason, the raw data on the monozygotic twin cohort cannot be deposited in a public database but can be made available through collaboration or upon request (contact Qihua Tan, qtan@health.sdu.dk).
All data was aligned to the GRCh38 assembly, unless otherwise specified. rDNA consensus sequences were obtained from GenBank (human KY962518.1, rat V01270.1).

Research involving human participants, their data, or biological material

Policy information about studies with human participants or human data. See also policy information about sex, gender (identity/presentation), and sexual orientation and race, ethnicity and racism.

Reporting on sex and gender	The initial discovery cohort in this study was of individuals that self-identified as male sex. Gender identity was not reported. The METSIM study was previously published GEO (GSE87893). This study included only individuals that self-identified as male sex, gender identity was not available for the analyses undertaken in this study. The monozygotic twin study included twin pairs of both self-reported sexes. This was confirmed by read mapping to the Y chromosome. As all analyses were within twin pairs and sample numbers were limited, sex was not included as a cofactor. Data from the 1000 genomes included individuals of both self-identified sexes. Sex was included as a cofactor in the GWAS analysis performed using this study. The rat liver study was previously published and performed exclusively using animals of female sex GEO (GSE157551). The data for the purified cell populations included donors of both sexes, as assigned in the original study (European Genome Archive (Dataset ID EGAD00001009789). Sex was not included as a cofactor in these analyses. Our findings are similar across both single sex and dual sex cohorts. We did not have enough power to included sex as a cofactor in analyses, with exception for the GWAS analysis.
Reporting on race, ethnicity, or other socially relevant groupings	The discovery cohort recruited at UCLH was of mixed ethnicity. Ethnic origin was self reported by participants as SEA (South East Asian), IND (indian), GBR (White British), SEU (South European), NEU (North European), NAF (Northern African), FEA (East African), UNC (unclassified), OTH (other/unknown). The cohort was too small to use ethnicity as a cofactor in the initial analyses. Ethnicity was used only in Figure S5 to show that the overall trend was not driven by one group. The METSIM study was previously published (PMID: 29566149). This study included only individuals that self-identified as Finnish, as provided in the original publication. Ethnicity was not used as a covariate in analysis of this cohort. The monozygotic twins study used here were originally from the Qingdao Twin Registry and reported by this third party to be of Chinese ancestry (PMID: 23182450). Ancestry was not included as a cofactor in these analyses. Data from the 1000 Genomes project included individuals of multiple ancestries as defined previously (PMID: 27638885). The 1000 Genomes populations were used as covariates in the GWAS performed in this study. The data from purified cell data (PMID: 36599988) contained ethnicity data, but this was not included as a covariate in our analyses due to limited sample numbers.
Population characteristics	The discovery cohort recruited at UCLH had a mean age of 36.4 +/- 5years. Based on metabolic measurements, no individuals had indicators or Type 2 Diabetes. No participants reported prior diagnoses or medication use. Study participants were selected based on their BMI (lean or obese). Anthropomorphic and phenotypic measurements are reflective this selection criteria. The METSIM study has previously been published (PMID: 29566149) and the population characteristics are described there-in. The primary characteristic of interest we compared in the present study were those individuals who were taking 1 or more medications daily versus those that were not. The monozygotic twin cohort was described in the original study (PMID: 31152155) to be free from medication and diagnosed diseases. Population variables for the 1000 Genomes data were limited to sex and ancestry, as discussed above (PMID: 27638885). Population data for the purified cell data was not considered (PMID: 36599988).
Recruitment	Participants were recruited as part of a prospective cohort study, the Dad's Health Study at University College Hospital London (see ethics approval below), to investigate the association between paternal metabolic health (including lean, overweight and obese men) and offspring birth weight. All participants provided written, informed consent. Whole blood samples were collected from participants recruited from University College London Hospital (UCLH) May 2016—March 2019. Participants were phenotyped with regards to BMI, waist circumference, systolic and diastolic blood pressure, blood lipids, fasting insulin and glucose levels and C-reactive protein (CRP). Two groups of participants were included; lean (BMI <25kg/m2) and obese (BMI >30kg/m2). Summary phenotypic data for each group is detailed in Table S1. BMI was determined with light clothing, by a trained researcher in the same visit at which blood samples were obtained. It is unlikely that self-selection bias has an effect as samples were chosen to be matched across the two groups based on other recorded parameters, such as age and self-reported ethnicity.
Ethics oversight	Ethical approval for the study was granted from the South East Coast—Surrey Research Ethics Committee on 28 September 2015 (REC reference number 15/LO/1437, IRAS project ID 164459). The study was also registered with the University College London Hospital Joint Research Office (Project ID 15/0548). All participants provided written, informed consent.

Note that full information on the approval of the study protocol must also be provided in the manuscript.

Field-specific reporting

Please select the one below that is the best fit for your research. If you are not sure, read the appropriate sections before making your selection.

☒ Life sciences ☐ Behavioural & social sciences ☐ Ecological, evolutionary & environmental sciences

For a reference copy of the document with all sections, see nature.com/documents/nr-reporting-summary-flat.pdf

Life sciences study design

All studies must disclose on these points even when the disclosure is negative.

Sample size	Sample sizes were chosen based on published epigenome-wide high throughput sequencing based studies available at the time. Please see PMID: 31152155, PMID: 26669700 and PMID: 27437034 as examples.
Data exclusions	Data was excluded if it exhibited low mapping efficiency, poor bisulfite conversion efficiency, extreme variation to data from the same study with regards to the number of uniquely aligned reads, or for the RRBS datasets exclusively, extreme variations in the global CpG methylation levels, as this could result from skewed genome representation.
Replication	We replicated the directional association between low rDNA CN and higher body mass across two independent human cohorts and an additional rat cohort. Interestingly, when the human replication cohort was divided based on medication status, only the unmedicated group replicated. This suggests that there are subgroups within this cohort.
Randomization	For the discovery cohort, samples were allocated to "Lean" if they had a BMI<25kg/m2 or "Obese" if they had BMI>30kg/m2 at the time of blood sampling. Samples were then further matched to have age at sampling differences between the two groups of <2 years and equal numbers of participants in each group. Other measurements available at this time were not utilised as cofactors as they are well-established to be influenced by BMI and therefore directly confounding of the variable of interest. Samples were only excluded downstream based on the quality control parameters. Randomization for all other studies utilised here are described in the relevant source publications.
Blinding	The experimenter was blinded at the time of sample selection and library preparation and was only unblinded after the sequencing data processing pipeline was complete. Unblinding was necessary at this stage to be able to interpret the data.

Reporting for specific materials, systems and methods

We require information from authors about some types of materials, experimental systems and methods used in many studies. Here, indicate whether each material, system or method listed is relevant to your study. If you are not sure if a list item applies to your research, read the appropriate section before selecting a response.

Materials & experimental systems

n/a	Involved in the study	
☒	☐	Antibodies
☒	☐	Eukaryotic cell lines
☒	☐	Palaeontology and archaeology
☒	☐	Animals and other organisms
☒	☐	Clinical data
☒	☐	Dual use research of concern
☒	☐	Plants

Methods

n/a	Involved in the study	
☒	☐	ChIP-seq
☒	☐	Flow cytometry
☒	☐	MRI-based neuroimaging

Plants

Seed stocks	*Report on the source of all seed stocks or other plant material used. If applicable, state the seed stock centre and catalogue number. If plant specimens were collected from the field, describe the collection location, date and sampling procedures.*
Novel plant genotypes	*Describe the methods by which all novel plant genotypes were produced. This includes those generated by transgenic approaches, gene editing, chemical/radiation-based mutagenesis and hybridization. For transgenic lines, describe the transformation method, the number of independent lines analyzed and the generation upon which experiments were performed. For gene-edited lines, describe the editor used, the endogenous sequence targeted for editing, the targeting guide RNA sequence (if applicable) and how the editor was applied.*
Authentication	*Describe any authentication procedures for each seed stock used or novel genotype generated. Describe any experiments used to assess the effect of a mutation and, where applicable, how potential secondary effects (e.g. second site T-DNA insertions, mosiacism, off-target gene editing) were examined.*
